# Evaluating Production of Cyclopentyl Tetraethers by Marine Group II *Euryarchaeota* in the Pearl River Estuary and Coastal South China Sea: Potential Impact on the TEX_86_ Paleothermometer

**DOI:** 10.3389/fmicb.2017.02077

**Published:** 2017-10-31

**Authors:** Jin-Xiang Wang, Wei Xie, Yi Ge Zhang, Travis B. Meador, Chuanlun L. Zhang

**Affiliations:** ^1^MARUM-Center for Marine Environmental Sciences, University of Bremen, Bremen, Germany; ^2^Department of Marine Sciences, University of Georgia, Athens, GA, United States; ^3^State Key Laboratory of Marine Geology, Tongji University, Shanghai, China; ^4^Department of Oceanography, Texas A&M University, College Station, TX, United States; ^5^Department of Ocean Science & Engineering, Southern University of Science and Technology, Shenzhen, China

**Keywords:** Marine Group II, *Euryarchaeota*, GDGTs, TEX_86_, ring index, South China Sea

## Abstract

TEX_86_ [TetraEther indeX of glycerol dialkyl glycerol tetraethers (GDGTs) with 86 carbon atoms] has been widely applied to reconstruct (paleo-) sea surface temperature. Marine Group I (MG-I) *Thaumarchaeota* were thought to be the primary source of GDGTs constituting the TEX_86_ formula; however, recent research has suggested that Marine Group II (MG-II) *Euryarchaeota* may also contribute significantly to the GDGT pool in the ocean. Little is known regarding the potential impact of MG-II *Euryarchaeota*-derived GDGTs on TEX_86_ values recorded in marine sediments. In this study, we assessed the relationship between distributions of GDGTs and MG-II *Euryarchaeota* and evaluated its potential effect on the TEX_86_ proxy. Lipid and DNA analyses were performed on suspended particulate matter and surface sediments collected along a salinity gradient from the lower Pearl River (river water) and its estuary (mixing water) to the coastal South China Sea (SCS, seawater). TEX_86_-derived temperatures from the water column and surface sediments were significantly correlated and both were lower than satellite-based temperatures. The ring index (RI) values in these environments were higher than predicted from the calculated TEX_86_-RI correlation, indicating that the GDGT pool in the water column of the PR estuary and coastal SCS comprises relatively more cyclopentane rings, which thereby altered TEX_86_ values. Furthermore, the abundance of MG-II *Euryarchaeota* 16S rRNA gene in the mixing water was two to three orders of magnitude higher than those observed in the river or seawater. Significant linear correlations were observed between the gene abundance ratio of MG-II *Euryarchaeota* to total archaea and the fractional abundance of GDGTs with cyclopentane rings. Collectively, these results suggest that MG-II *Euryarchaeota* likely produce a large proportion of GDGTs with 1–4 cyclopentane moieties, which may bias TEX_86_ values in the water column and sediments. As such, valid interpretation of TEX_86_ values in the sediment record, particularly in coastal oceans, should consider the contribution from MG-II *Euryarchaeota*.

## Introduction

TEX_86_ is a popular temperature proxy applied in paleoclimatological studies, which is based on the relative distribution of cyclopental rings among isoprenoid glycerol dialkyl glycerol tetraether (GDGT; Figure S1) lipids produced by archaea in marine and terrestrial environments (see review by Schouten et al., 2013). Global core-top calibrations of TEX_86_ values were empirically correlated with the annual mean sea surface temperature (SST; Schouten et al., 2002; Kim et al., 2008, 2010). However, mounting evidence indicates anomalies of TEX_86_-derived SST in coastal seas and the open ocean, which have been attributed to multiple inputs of GDGTs from terrestrial (e.g., Weijers et al., 2006) or bathypelagic sources (e.g., Lee et al., 2008), as well as production in marine sediments (e.g., Liu X. L. et al., 2011).

A great deal of effort has been made to assess TEX_86_ accuracy in marine and lake sediments. For example, application of the TEX_86_ proxy is cautioned under the following circumstances: a branched and isoprenoid tetraether (BIT) index > 0.2 (Zhu et al., 2011), a ratio of GDGT-2/crenarchaeol > 0.4 (Weijers et al., 2011a), a Methane Index > 0.5 (Zhang et al., 2011), a ratio of GDGT-0/crenarchaeol > 2 (Blaga et al., 2009), or when %GDGT-2 > 45 (Sinninghe Damsté et al., 2012). Recently, based on an assessment of the relationship between the weighted average number of cyclopentane rings in GDGTs (ring index, RI) and published TEX_86_ data from core-top sediments, Zhang et al. (2016) established a significant correlation between TEX_86_ and RI [RI = 3.32 × (TEX_86_)^2^ − 0.77 × TEX_86_ + 1.59; ±2σ ~ 0.3]. This relationship was expected and reflects the physiological response of marine archaea to synthesize GDGTs with more rings (higher RI values) at higher temperatures (higher TEX_86_ values). Deviations from this relationship suggest that temperature is not a dominant factor governing GDGT ring distribution, or, alternatively, the relationship between GDGT ring distribution and temperature is different from the modern analog as defined by the global core-top dataset.

The TEX_86_-related GDGTs (GDGTs-1, -2, -3 and the crenarchaeol regioisomer) in the water column are thought to primarily derive from the Marine Group I (MG-I) *Thaumarchaeota* (e.g., Schouten et al., 2008; Pitcher et al., 2011a,b), as it is one of the dominant groups of planktonic archaea in the ocean (Karner et al., 2001). In particular, crenarchaeol, containing one cyclohexane and four cyclopentane moieties, is accepted as a specific biomarker for MG-I *Thaumarchaeota* (Sinninghe Damsté et al., 2002; Schouten et al., 2008). Marine Group II (MG-II) *Euryarchaeota* are another group of planktonic archaea that predominantly inhabit coastal water and (near) surface waters of the open ocean (e.g., DeLong, 1992; Galand et al., 2010; Hugoni et al., 2013). Recently, this cosmopolitan group was also invoked as another major source of GDGTs (including crenarchaeol) in the ocean (Lincoln et al., 2014a), which supports an earlier hypothesis about GDGT-producing MG-II *Euryarchaeota* (Turich et al., 2007). However, concrete evidence of crenarchaeol production by MG-II is lacking due to the inability to obtain a pure culture or isolate, and the exact composition of GDGTs produced by these organisms remains uncertain (Lincoln et al., 2014b; Schouten et al., 2014).

To further evaluate the relationship between MG-II *Euryarchaeota* and the distribution of GDGTs, we quantified the abundance of MG-II 16S rRNA gene, determined the distribution of GDGT core and intact polar lipids (CL and IPL, respectively), and assessed TEX_86_ and Ring Index (RI) values from suspended particulate matter and surface sediments collected from the lower Pearl River (PR) and its estuary to the coastal South China Sea (SCS). Our results provide a mechanistic explanation for deviations of the TEX_86_ paleothermometer and have important implications for the sources of GDGTs in marine environments and probing past changes in global climate.

## Materials and methods

### Sample collection

**Figure 1:**
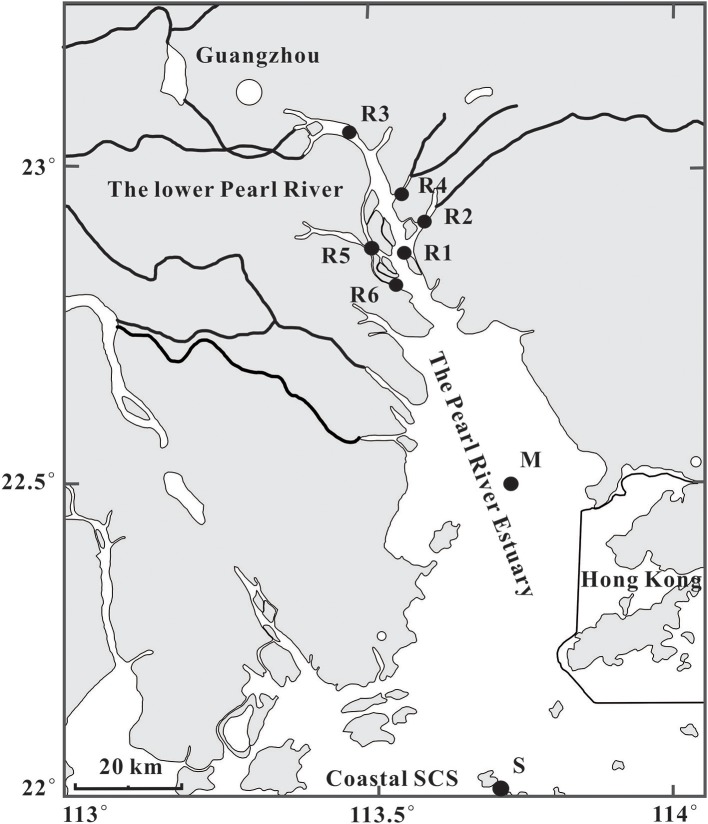
Map showing locations of sites (dark circles) in the lower Pearl River (PR), the PR estuary, and coastal South China Sea (SCS). R, River water, which is followed by the station #1–6; M, mixing water; S, seawater. Station S includes four water layers (surface, subsurface, middle, and bottom). Station M includes three water layers (surface, middle, and bottom). The surface water at station M was also collected during the high tide-, slack tide-, and low tide-periods. Stations R1 and R2 include two water layers (surface and bottom). Surface sediments were collected at each sampling site.

Sampling locations and other information for suspended particulate matter (SPM) and surface sediments are shown in Figure 1 and Table 1. SPM samples (*n* = 18) and surface sediments (*n* = 8) were collected along a salinity gradient from the lower Pearl River and its estuary to the coastal South China Sea in the summer of 2011. SPM samples were collected from the surface (stations R1–R6) and the bottom (stations R1 and R2) water in the lower Pearl River, from three water depths (surface, middle, and bottom) and during three tidal periods (high tide, slack tide, and low tide) at station M located in the PR estuary, and at four water depths (surface, subsurface, middle, and bottom) at station S in the seawater of the coastal SCS (Figure 1). The depth of the sampling layers in the water column is given in Table 1. About 4–103 liters of water were filtered onto combusted (450°C, overnight) glass-fiber filters (Whatman GF/F, 0.7 μm, 142 mm diameter) using an *in situ* submersible pump. The pH, temperature, salinity, and depth were determined *in situ* by a Horiba instrument (W-20XD, Kyoto, Japan; Table 1). Surface sediments (top ca. 10 cm) were collected at all stations (Figure 1; Table 1) using a grab sampler. All samples were frozen immediately in liquid nitrogen and kept at −80°C in the laboratory before analysis.

**Table 1:** Basic information, abundance of isoprenoid GDGTs, TEX_86_, Ring Index, and 16S rRNA gene abundances for suspended particulate matter (SPM) in the water column and surface sediments collected from the lower Pearl River, the Pearl River estuary, and coastal northern South China Sea.

**Sample ID^a^**	**Longitude (E)**	**Latitude (N)**	**Sampling date (mm/dd/yyyy)**	**Depth^b^ (m)**	**Temp. (°C)**	**Sal**.	**pH**	**isoGDGTs (ng/l)^c^**			**TEX**_**86**_			**Ring Index (RI_2_)^d^**			**Archaeal 16S (copies/l)**	**MG-II 16S (copies/l)**
								**CL**	**Total-IPL**	**Phospho-IPL**	**CL**	**Total-IPL**	**Phospho-IPL**	**CL**	**Total-IPL**	**Phospho-IPL**		
**RIVER WATER SPM**																		
R1_sur	113°34.249′	22°52.647′	06/21/2011	1.5	29.7	0.2	7.25	188.6	201.5	16.7	0.59	0.50	0.57	0.19	0.15	0.12	2.1E+09	3.0E+05
R1_bott	113°34.249′	22°52.647′	06/21/2011	6.0	29.6	0.2	7.25	221.3	128.1	8.9	0.60	0.56	0.54	0.16	0.12	0.15	3.6E+08	1.2E+03
R2_sur	113°36.680′	22°56.338′	06/21/2011	1.5	29.0	0.1	6.95	64.8	82.5	6.8	0.56	0.42	0.49	0.16	0.15	0.11	–^e^	–
R2_bott	113°36.680′	22°56.338′	06/21/2011	6.0	29.0	0.1	6.90	266.6	426.5	27.9	0.60	0.45	0.38	0.14	0.10	0.12	–	–
R3	113°28.726′	23°04.339′	06/22/2011	1.5	29.4	0.1	7.46	79.8	150.2	8.0	0.57	0.41	0.38	0.07	0.09	0.14	–	–
R4	113°33.507′	22°58.409′	06/22/2011	1.5	29.6	0.2	7.28	19.1	30.6	3.3	0.63	0.57	0.37	0.26	0.13	0.20	–	–
R5	113°29.941′	22°53.588′	06/22/2011	1.5	28.5	0.1	7.28	20.0	30.4	2.0	0.61	0.48	0.35	0.37	0.40	0.34	–	–
R6	113°33.088′	22°44.811′	06/22/2011	1.5	27.8	0.1	6.92	49.1	21.8	1.2	0.62	0.68	0.53	0.15	0.19	0.25	–	–
**MIXING WATER SPM**																		
M_lt	113°45.098′	22°27.206′	06/18/2011	1.5	–	–	–	25.9	105.0	2.4	0.66	0.55	0.53	0.52	0.47	0.43	2.0E+07	6.1E+06
M_st	113°45.098′	22°27.206′	06/18/2011	1.5	–	–	–	35.3	97.3	1.4	0.58	0.65	0.60	0.36	0.61	0.61	1.3E+09	3.1E+08
M_ht	113°45.098′	22°27.206′	06/18/2011	1.5	–	–	–	21.9	86.8	2.8	0.59	0.56	0.55	0.43	0.46	0.38	6.9E+07	1.2E+07
M_sur	113°45.098′	22°27.206′	06/18/2011	1.5	28.7	11.1	8.03	18.9	67.0	1.9	0.58	0.60	0.63	0.36	0.44	0.65	1.5E+09	5.7E+08
M_mid	113°45.098′	22°27.206′	06/18/2011	5.0	28.3	15.6	7.93	102.6	73.4	5.2	0.61	0.64	0.59	0.35	0.47	0.36	6.0E+09	7.9E+08
M_bott	113°45.098′	22°27.206′	06/18/2011	9.0	27.6	23.0	7.89	107.4	76.7	6.0	0.56	0.60	0.58	0.30	0.42	0.38	5.1E+09	1.6E+09
**SEA WATER SPM**																		
S_sur	113°70.448′	22°05.165′	06/15/2011	1.5	29.6	29.5	8.63	1.1	0.7	0.1	0.52	0.65	0.58	0.28	0.39	0.22	1.0E+05	4.8E+03
S_subs	113°70.448′	22°05.165′	06/15/2011	5.0	29.5	29.7	8.64	2.1	1.1	0.1	0.56	0.63	0.58	0.27	0.33	0.24	3.1E+06	5.3E+05
S_mid	113°70.448′	22°05.165′	06/15/2011	10.0	28.6	31.7	8.45	12.6	13.5	0.6	0.49	0.55	0.50	0.20	0.24	0.23	9.8E+04	4.4E+03
S_bott	113°70.448′	22°05.165′	06/15/2011	18.0	25.4	33.7	7.92	21.4	9.6	0.7	0.53	0.59	0.49	0.20	0.39	0.21	1.3E+08	1.9E+07
**SEDIMENT**																		
Sedi-R1	113°34.249′	22°52.647′	06/21/2011	8.0	–	–	7.46	461.8	142.8	9.5	0.58	0.37	0.30	0.27	0.38	0.41	–	–
Sedi-R2	113°36.680′	22°56.338′	06/21/2011	7.0	–	–	7.68	687.5	289.2	17.0	0.56	0.35	0.31	0.23	0.22	0.26	–	–
Sedi-R3	113°28.726′	23°04.339′	06/22/2011	9.0	–	–	7.29	800.3	233.5	19.6	0.57	0.43	0.36	0.15	0.23	0.30	–	–
Sedi-R4	113°33.507′	22°58.409′	06/22/2011	7.0	–	–	7.50	881.0	149.7	9.7	0.58	0.51	0.38	0.33	0.75	0.62	–	–
Sedi-R5	113°29.941′	22°53.588′	06/22/2011	8.0	–	–	7.58	621.7	84.2	7.3	0.62	0.52	0.37	0.33	0.66	0.43	–	–
Sedi-R6	113°33.088′	22°44.811′	06/22/2011	8.0	–	–	7.66	120.2	103.8	6.2	0.67	0.35	0.42	0.42	0.65	0.74	–	–
Sedi-M	113°45.098′	22°27.206′	06/18/2011	12.0	–	–	7.60	200.4	67.7	2.3	0.62	0.52	0.50	0.38	0.69	0.42	–	–
Sedi-S	113°70.448′	21°95.165′	06/15/2011	20.0	–	–	7.40	5003.6	267.3	20.8	0.50	0.64	0.50	0.19	0.73	0.34	–	–

*Basic information includes location, sampling date, water depth, temperature (Temp.), salinity (Sal.), and pH*.

a *R, River (the lower Pearl River), which is followed by the station numbers; sur and bott represent surface water and bottom water, respectively. M, Mixing water (the Pearl River estuary); lt, low tide; st, slack tide; ht, high tide; mid means middle layer water. S, Sea water (northern South China Sea); subs represents subsurface water*.

b *For the SPM samples collected from the water column, the depth is referred to the sampling water depth; For the sediments, the depth indicates the river water depth*.

c *CL, core lipids; total-IPL, intact polar lipid (IPL) derived core lipids upon acid (H) hydrolysis; phospho-IPL, IPL-derived core lipids derived upon base (OH) hydrolysis*.

d RI_2_ = ([GDGT-1] + 2^*^[GDGT-2] + 3^*^[GDGT-3] + 4^*^[GDGT-4] + 4^*^[Cren.iso])/100.

e *-, data are not available or not examined*.

### GDGT extraction and separation

The SPM samples (*n* = 18) and surface sediments (*n* = 8) were freeze-dried and extracted using a modified Bligh and Dyer method (Bligh and Dyer, 1959); the separation of core lipids and intact polar lipids followed the procedure described in Weijers et al. (2011b). Briefly, the total lipid extract (TLE) was obtained by ultrasonic extraction (10 min each, 6 times) of SPM (1 filter) or sediments (5 g) with a single-phase solvent mixture of methanol, dichloromethane (DCM), and phosphate buffer (2:1:0.8, v/v/v; pH 7.4). The TLE was separated over an activated silica gel column eluted with n-hexane/ethyl acetate (1:1, v/v) and methanol for CL and IPL, respectively. For GDGT quantification, a known amount of an internal C46 GDGT standard was added into the CL fraction or IPL fraction (Huguet et al., 2006). The CL fraction was directly measured. The IPL fraction was determined by measuring the CL after cleavage of the polar head groups via acid or base hydrolysis (Pitcher et al., 2009; Weijers et al., 2011b). Briefly, 1/3 IPL fraction (non-hydrolyzed IPL fraction) was directly condensed; another 1/3 IPL fraction was hydrolyzed (2 h) in 1.5 N HCl in methanol, which was called the acid-hydrolyzed IPL fraction (total IPL). DCM and MilliQ water were added, and the DCM fraction was collected (repeated 4 times). The DCM fraction was rinsed (6 times) with MilliQ water in order to remove acid and dried under N_2_ gas. The last 1/3 IPL fraction was subjected to base hydrolysis (2 h) in a 1N KOH in methanol/H_2_O mixture (95:5, v/v), which was called the base-hydrolyzed IPL fraction (phospho IPL). Together with the condensed CL fraction, the CL fraction, two fractions of IPL-derived core lipids and non-cleaved IPL fraction were dissolved in n-hexane/isopropanol (99:1, v/v), and filtered using PTFE filters (pore diameter of 0.45 μm). The acid-hydrolyzed IPL reflected total IPL, which, prior to hydrolysis, were attached to both phosphatidyl and glycosidic head groups; The base-hydrolyzed IPL represented IPLs with phosphatidyl head groups only (phospho IPL; Weijers et al., 2011b). Analysis of the non-hydrolyzed IPL fraction was performed to determine any carryover of CL into the IPL fraction.

### GDGT analysis

GDGTs from all treatments were analyzed using high performance liquid chromatography-atmospheric pressure chemical ionization-tandem mass spectrometry (HPLC-APCI-MS/MS), which was performed as described by Zhang et al. (2012), using an Agilent 1200 LC equipped with an automatic injector and coupled to a QQQ 6460 MS; peaks were evaluated using Mass Hunter LC-MS manager software. Separation was achieved using a Prevail Cyano column (2.1 × 150 mm, 3 μm; Alltech Deerifled, IL, USA) with n-hexane (solvent A) and a mixture of n-hexane/isopropanol 90/10 (v/v; solvent B). The (M+H)^+^ ions of each core isoprenoid GDGT (m/z 1,302, 1,300, 1,298, 1,296, 1,294, 1,292) were monitored via selected ion monitoring (SIM) mode (Schouten et al., 2007).

### GDGT-based indices

Indices based on the fractional abundance of GDGTs were calculated as follows:

(1) $$\begin{array}{l} {\text{TEX}}_{86}=([GDGT-2]+[GDGT-3]+[Cren.iso])\\ \;/\;([GDGT-1]\text{​}+\text{​}[GDGT\text{​}-\text{​}2]\text{​}+\text{​}[GDGT\text{​}-\text{​}3]\\ \;+\;[Cren.iso])\; \end{array}$$

(Schouten et al., 2002)

(2) $$\begin{array}{l} \text{Ring }{\text{Index}}_{1}({\text{RI}}_{1})=([GDGT-1]+2\;\times [GDGT-2]+3\\ \;\times \;[GDGT-3]+4\;\times [Cren.]+4\;\\ \;\times \;[Cren.iso])/100 \end{array}$$

(Zhang et al., 2016)

(3) $$\begin{array}{l} \text{Ring Index}({\text{RI}}_{2})=([GDGT-1]+2\;\times [GDGT-2]+3\\ \;\times \;[GDGT-3]+4\;\times [GDGT-4]+4\\ \;\times \;[Cren.iso])/100 \end{array}$$

with the GDGT numbers corresponding to the GDGT structures in Figure S1. Note that RI_2_ was originally developed for the current study and modified from RI_1_ (Zhang et al., 2016), in which the fractional abundance of crenarchaeol was replaced by GDGT-4 in order to eliminate the influence of crenarchaeol on weighted average number of cyclopentane rings in GDGTs.

### DNA extraction and the quantitative polymerase chain reaction (qPCR)

The SPM samples (*n* = 12) from station R1 (river water), station M (mixing water), and station S (seawater) were selected for the DNA analysis. The frozen filters were washed 3 times by phosphate buffered saline (pH 7.4). The supernatants were centrifuged under 11,000 *g* for 10 min. The DNA was extracted following the protocol of FastDNA SPIN Kit. The DNA samples were dissolved with a final dilution in 100-μL deionized water and preserved at −80°C until further processing. The DNA concentrations were quantified in duplicate with a Nano-Drop spectrophotometer (Thermo Fisher Scientific Inc., Wilmington, DE, USA). The quantitative PCR primers were Arch_334F (5′ACGGGGCGCAGCAGGCGCGA3′)/Arch_ 518R (5′ATTACCGCGGCTGCTGG3′) for total archaeal 16S gene quantification (Bano et al., 2004), GII-554-f (5′GTCGMTTTTATTGGGCCTAA3′), and Eury806-r (5′CACAGCGTTTACACCTAG3′) for MG-II *Euryarchaeota* 16S gene quantification (Massana et al., 1997; Teira et al., 2004). The qPCR analysis was performed at 95°C for 30 s and 40 cycles at 94°C for 30 s, 55°C for total archaea and 53°C for MG-II *Euryarchaeota* for 30 s and 68°C for 1 min. Triplicate measurements were run for each sample and standard.

PCR bands of 16S rRNA gene and MG-II gene were amplified from SPM samples in Station M. They were recovered by a Gel Extraction Kit (omega) and sequenced on the 3730-sequencing platform. The sequences were annotated as the corresponding target genes, which demonstrated the specificity of the chosen qPCR primers. A dilution series of purified DNA from those positive clones were used as standards. A melting curve analysis was performed to demonstrate that the fluorescent signal obtained in a given reaction was consistent with the expected profile for specific PCR products. The *R*^2^ values of standard curves were >0.99. The efficiency of each qPCR was between 87 and 99%.

### Amplicon sequencing of the archaeal 16S rRNA gene

SPM samples collected in river water, mixing water and seawater were selected to conduct MiSeq pyrosequencing targeting the archaeal 16S rRNA gene. In contrast to the qPCR primers, these primers targeted longer sequences to increase the precision of phylogenetic analysis. The primers were Arch_344F (5′ACGGGGCGCAGCAGGCGCGA3′) and Arch_915R (5′GTGCTCCCCCGCCAATTCCT3′; Gantner et al., 2011). The MiSeq sequencing was conducted on the MiSeq platform (2 × 250 PE, Illumina) at the Shanghai Personalbio Biotechnology (Shanghai, China). Mothur (version 1.29.2; Schloss et al., 2009) was applied to filter the raw pyrosequencing data. The selected sequences were analyzed using the QIIME standard pipeline (Caporaso et al., 2010). Taxonomy was assigned according to the Ribosomal Database Project (RDP) classifier 2.2 (minimum confidence of 80%; Cole et al., 2009). The GenBank accession numbers are PRJNA38421 for those archaeal 16S rRNA genes.

### Satellite-derived surface water temperature (SWT)

The satellite-derived SWT was determined with a spatial resolution of 4 km from the NOAA advanced very-high-resolution radiometer (AVHRR; version 5.2; http://www.nodc.noaa.gov/SatelliteData/pathfinder4km/). The June mean SWT was obtained from the daily averaged values of 30 days in June 2011 (sampling month). The annual mean SWT and winter mean SWT represented 8-year mean values of annual mean temperature (2004–2011) and monthly mean temperature (December–February), respectively, as the surface sediment (top ca. 10 cm) collected in this study might represent a deposition of 6–10 years, based on an estimation from Strong et al. (2012).

## Results and discussion

### TEX_86_-derived temperature and ring index

**Figure 2:**
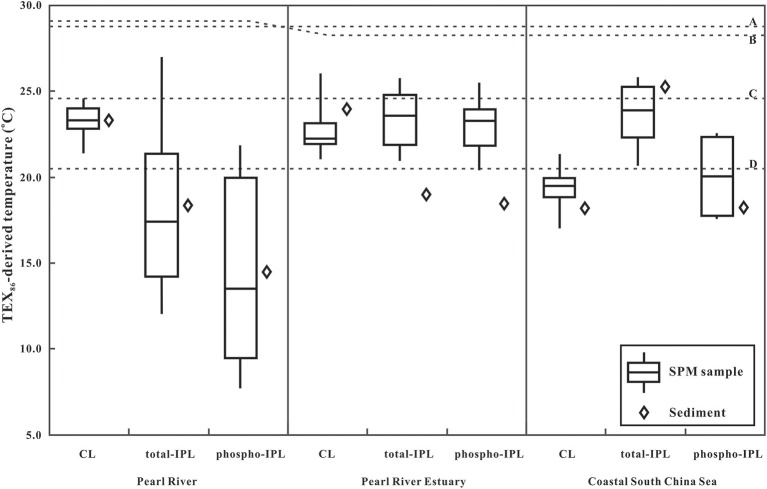
Mean values of TEX_86_-derived temperatures in SPM and surface sediments from the lower Pearl River (R), the PR estuary (M), and coastal SCS (S). CL, core lipids; total-IPL, intact polar lipid based upon acid hydrolysis; phosphor-IPL, intact polar lipid based upon base hydrolysis. Dashed lines A, June mean surface water temperature (SWT; 28.4 ± 0.07°C); dashed line B, *in situ* instrumental temperature (29.1°C, in the river water; 28.2°C in the mixing water and seawater); dashed line C, annual mean SWT (24.71 ± 0.11°C); dashed line D, winter SWT (20.54 ± 0.10°C).

The TEX_86_-derived temperature was calculated based on the calibration of Kim et al. [SST = 68.4 × log (TEX_86_) + 38.6; (Kim et al., 2010)]. The CL-TEX_86_ temperatures derived from either the SPM or the surface sediments were close to the satellite-based annual mean SWT in the river water and mixing water, station R and M, respectively; whereas CL-TEX_86_ temperatures for the seawater station S were lower than the winter mean SWT (Figure 2). The correspondence between the CL-TEX_86_ temperatures derived from SPM and sediment samples (*R*^2^ = 0.70, *P* < 0.01) along the salinity gradient indicated that the TEX_86_ signal in the sediment predominantly reflected archaea from the water column, which is consistent with previous studies in the PR estuary (Wang et al., 2015) and other coastal settings (Herfort et al., 2006; Zell et al., 2014).

Total IPL-TEX_86_ temperatures in the water column were consistently lower than both the June mean SWT and *in situ* measurements, although the sampling season was summer (Figure 2). In the mixing water and seawater, the phospho IPL-TEX_86_ was lower than the total IPL-TEX_86_ whereas it was very close to the CL-TEX_86_ (Figure 2; Table 1). Since phosphatidyl head groups can be degraded faster than the glycosidic head groups, the phospho IPL is considered to be a better reflection of the living microorganisms (Harvey et al., 1986; Schouten et al., 2010), which may explain the deviation between these IPL pools. Relatively rapid conversion of phospho IPL to CL may result in more similar ring distributions and thus TEX_86_ values between phosphor IPL and CL. Furthermore, in the same study area, the variability in TEX_86_ was suggested to be due to changes in archaeal community composition in the water column, in which the unusually low TEX_86_-derived temperature in the coastal SCS was speculated to be linked to MG-II *Euryarchaeota* (Wang et al., 2015).

**Figure 3:**
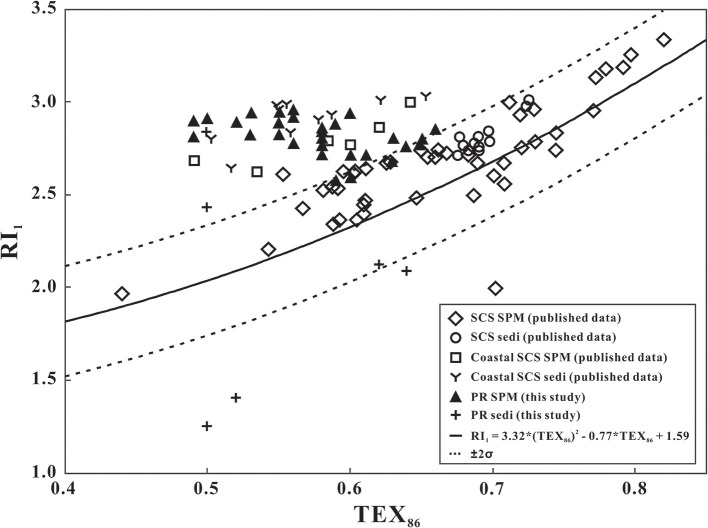
TEX_86_ of the PR SPM samples and PR surface sediments plotted against RI_1_. The solid curve represents the RI-TEX_86_ calibration from Zhang et al. (2016). The SCS SPM/sediments and coastal SCS SPM/sediments are from Wei et al. (2011), Ge et al. (2013), Zhang et al. (2013), and Wang et al. (2015).

Since TEX_86_ can be influenced by factors other than temperature, the ring index was proposed to evaluate the accuracy of TEX_86_ in marine sediments (Zhang et al., 2016). Here, CL-, phosphor IPL-, and total IPL-TEX_86_ values were plotted against RI_1_ (Equation 2; Figure 3) using data derived from the SPM and surface sediment samples collected during the current and previous studies (Wei et al., 2011; Ge et al., 2013; Zhang et al., 2013; Wang et al., 2015). Most SPM and surface sediment samples from the open South China Sea plotted within the RI_1_-TEX_86_-confined zone [RI_1_ = 3.32 × (TEX_86_)^2^ − 0.77 × TEX_86_ + 1.59, ±2σ ~ 0.3; (Zhang et al., 2016)], whereas the majority of samples from coastal SCS and the PR estuary fell above the calibration zone (Figure 3), exhibiting higher ring index values than those from the open SCS. This implies that the GDGT pool in the water column of the PR estuary and coastal SCS comprised relatively more cyclopentane rings than predicted from the measured SWT. If it is assumed that GDGTs produced only by *Thaumarchaeota* underpin the relationship between SWT and TEX_86_ (e.g., Schouten et al., 2002), then it follows that either *Thaumarchaeota* in the PR estuary respond differently to temperature than marine strains, or there is another source of cycloalkyl-containing GDGTs in the estuary.

**Figure 4:**
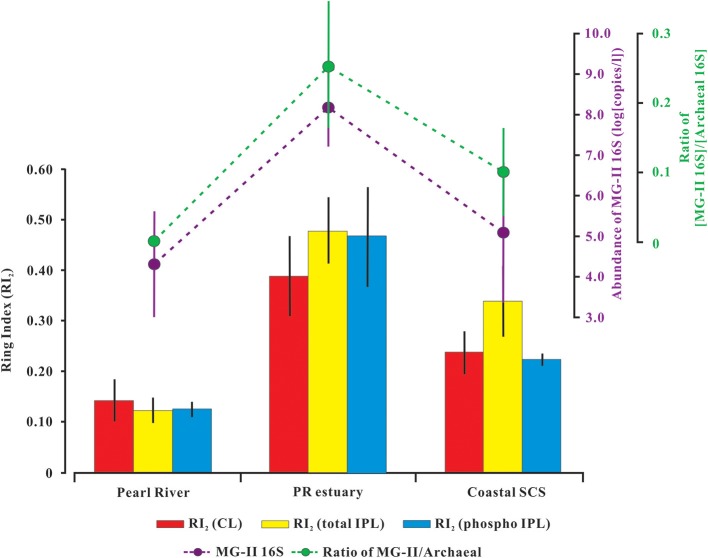
Distribution of the mean values of Ring Index (RI_2_) compared with the abundance of MG-II *Euryarchaeota* 16S rRNA gene and the gene abundance ratio of MG-II *Euryarchaeota* to total archaea along the salinity gradient from the river water to seawater. RI (Equation 3) was calculated from CL (red bars), total-IPL (yellow bars), and phosphor-IPL (blue bars). Details are shown in Table 1.

To further assess the distribution of RI values in the SPM and to explore other contributor(s) to the cyclopentyl GDGT pool in the study area, mean values of CL-, total IPL-, and phospho IPL-ring index were examined (Figure 4). Note that crenarchaeol, a biomarker for MG-I *Thaumarchaeota*, was excluded from the ring index calculation (RI_2_, Equation 3) in order to limit its overwhelming influence on the index. The re-defined RI_2_ equation is more sensitive to the variation of cyclopentane-containing GDGTs that might be contributed by other archaea. Compared with the river water and seawater, the highest RI_2_ value for either CL (avg. 0.39 ± 0.08) or IPL (avg. 0.48 ± 0.07 for total IPL; avg. 0.47 ± 0.10 for phospho IPL) occurred at station M in the mixing water (Figure 4; Table 1), suggesting that the PR estuary (station M) appeared to be a hot spot of production of GDGTs with cyclopentane moieties. Further confirmation came from the comparison of %GDGT 1–4 in different water settings (Table S1), which showed that the sum of the fractional abundances of the GDGTs with 1–4 cyclopentane moieties in the mixing water was significantly higher than that in the seawater or river water. In the mixing water station, the mean values of total IPL-RI and phospho IPL-RI were not significantly different; both were higher than the CL-RI (Figure 4). In the seawater station, however, the total IPL-RI (0.34 ± 0.07) was more elevated than the phospho IPL-RI (0.22 ± 0.01) and the CL-RI (0.24 ± 0.04; Figure 4). This ring index distribution pattern at station S corresponded to the TEX_86_-temperature distribution in the SPM and sediment samples (Figure 2), suggesting that cyclopentane-containing GDGTs altered the TEX_86_ record in the water column, and the vertical transportation of GDGTs from the water column appeared to be a predominant source to the sediment.

### Relationship between MG-II *Euryarchaeota* and cyclopentane-containing GDGTs

Previous studies reported that a significant proportion of MG-II *Euryarchaeota* was diversely present in the estuarine and coastal regions, including the Pearl River estuary (Liu et al., 2014; Wang et al., 2015), the Yangtze River estuary (Liu M. et al., 2011), and the Jiulong River estuary (Hu et al., 2015). In this study, the MG-II *Euryarchaeota* 16S rRNA gene averaged 5.4 ± 5.9 × 10^8^ copies L^−1^ (*n* = 6) at the mixing water station, which was two to three orders of magnitude higher than that in river water station (avg. 1.5 ± 2.1 × 10^5^ copies L^−1^, *n* = 2) and seawater station (avg. 4.9 ± 9.4 × 10^6^ copies L^−1^, *n* = 4; Figure 4). Considering the heterotrophic life style of MG-II, which have been demonstrated by the former genetic analysis (Iverson et al., 2012; Li et al., 2015; Martin-Cuadrado et al., 2015) and cultivation experiment (Orsi et al., 2015, 2016), the high abundances of MG-II in the mixing water station seem to be due to the high phototrophs that enhanced by nutrients input from upper river (Gan et al., 2014).

The ratio of MG-II *Euryarchaeota* 16S rRNA gene abundance to total archaeal 16S rRNA gene abundance ([MG-II 16S]/[Archaea 16S]) in mixing water (avg. 0.25 ± 0.08, *n* = 6) was significantly higher than in the seawater (avg. 0.10 ± 0.004, *n* = 4; *P* < 0.01), whereas it was negligible (avg. <0.0001) in the river water (Figure 4). This observation was also supported by pyrosequencing analysis, which exhibited a linear correlation with the qPCR-based ratio of [MG-II]/[Archaea 16S] (Figure S2; Table S2). These results further confirmed that the PR estuary (mixing zone, salinity avg. 16.6) provided a habitat to sustain a natural enrichment of planktonic MG-II *Euryarchaeota*.

**Figure 5:**
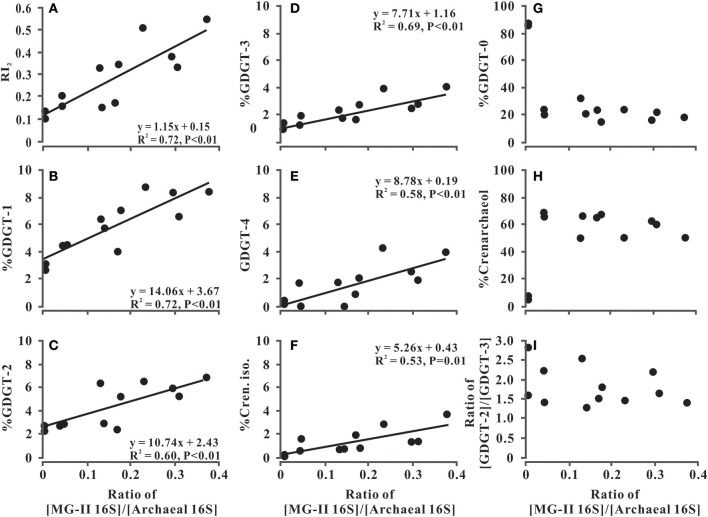
RI_2_ **(A)**, fractional abundance of GDGTs **(B–E, G)** and Crenarchaeol and isomer (**H,F**, respectively), and the ratio of GDGT-2 to GDGT-3 **(I)** vs. the ratio of the MG-II *Euryarchaeota* 16S rRNA genes to the total archaeal 16S rRNA genes. The black points represent SPM samples collected from the lower Pearl River, the PR estuary, and the coastal SCS.

The presence of (more labile) phospho IPL-GDGTs implied *in situ* production of isoprenoidal GDGTs in the water column along the entire salinity gradient from the Pearl River to coastal SCS. The elevated phospho IPL-RI in the mixing water additionally suggests that higher relative proportions of GDGTs with 1–4 cyclopentane moieties were produced in the PR estuary. A linear regression analysis confirmed the positive relationship between phospho IPL-derived RI and the [MG-II 16S]/[Archaea 16S] ratio in the water column along the salinity gradient (*R*^2^ = 0.72, *P* < 0.01; Figure 5A). Therefore, it is reasonable to hypothesize that MG-II *Euryarchaeota* preferentially synthesized GDGTs with 1–4 cyclopentane moieties in this region. Moreover, production of GDGTs-1 to -4 by MG-II *Euryarchaeota* could represent the missing source needed to explain the elevated value of RI and deviation from the RI-TEX_86_ relationship driven by the preservation of *Thaumarchaeota* GDGTs in global core-top sediments.

To further constrain the relationship between MG-II *Euryarchaeota* and cyclopentane-containing GDGTs, linear regression analysis was conducted between the fractional abundance of GDGTs and the [MG-II 16S]/[Archaea 16S] ratio in the SPM along the salinity gradient. Results exhibited a significantly positive linear correlation between the [MG-II 16S]/[Archaea 16S] ratio and the fractional abundance of phospho IPL-based GDGT with cyclopentane moieties (Figures 5B–F). In contrast, we observed no correlation between the [MG-II 16S]/[Archaea 16S] ratio and the fractional abundances of phospho IPL-based GDGT-0 or crenarchaeol (Figures 5G,H; Table 2). Similar trends of the linear correlations were also observed between the [MG-II 16S]/[Archaea 16S] ratio and the CL- and total IPL-GDGTs with 1–4 cyclopentane moieties, with a less significant correlation between the [MG-II 16S]/[Archaea 16S] ratio and the total IPL-based crenarchaeol regioisomer (Table 2). Although MG-II *Euryarchaeota* were suggested to be an alternative source of crenarchaeol in the ocean (Lincoln et al., 2014a), our study showed an absence of a significant correlation between the distribution of MG-II *Euryarchaeota* and crenarchaeol (Figure 5H). However, members of MG-II *Euryarchaeota* living in the North Pacific Subtropical Gyre (i.e., those targeted by Lincoln et al., 2014a) may be different from those living in the coastal zone of the PR estuary. This is consistent with the phylogenetic distribution of MG-II reported by Wang et al. (2015), which showed diverse groups of MG-II living in this region.

**Table 2:** Regression analysis between the ratio of MG-II 16S rRNA genes to Archaeal 16S rRNA genes vs. the fractional abundance of GDGTs, ring index (RI_2_), and the ratio of GDGT-2 to GDGT-3.

	**[MG-II/Archaea] vs. CL^a^**		**[MG-II/Archaea] vs. total-IPL**		**[MG-II/Archaea] vs. phospho-IPL**	
	***R*^2^**	***P*-value**	***R*^2^**	***P*-value**	***R*^2^**	***P*-value**
%GDGT-0	0.37	0.04	0.44	0.02	0.40	0.03
%GDGT-1	0.50	0.01	0.58	0.00	0.72	0.00
%GDGT-2	0.43	0.02	0.47	0.01	0.60	0.00
%GDGT-3	0.33	0.05	0.51	0.01	0.69	0.00
%GDGT-4	0.43	0.02	0.46	0.02	0.58	0.00
%Cren.	0.25	0.10	0.37	0.03	0.21	0.13
%Cren.iso	0.56	0.01	0.11	0.80	0.53	0.01
RI_2_	0.49	0.01	0.50	0.01	0.72	0.00
GDGT 2/3	0.13	0.25	0.30	0.07	0.11	0.29

a *[MG-II/Archaea], the 16S rRNA gene ratio of MG-II to archaeal. CL, core lipids; total-IPL, intact polar lipids derived upon acid hydrolysis; phospho-IPL, intact polar lipids derived upon base hydrolysis*.

By comparison of the *R*^2^ values and slopes of the regression equations (Figures 5B–F), GDGT-1 exhibited not only the strongest correlation with MG-II *Euryarchaeota* in the study area, but also largest relative enrichment (i.e., slope of 14.06). If MG-II *Euryarchaeota* preferentially synthesized GDGT-1, additional contribution of GDGT-1 to the water column of the PR estuary and the coastal SCS would be reflected by an increase in RI values and a substantial decrease in TEX_86_ values. Offsets in TEX_86_ values have been similarly proposed to result from the decreased ratio of GDGT-2 to GDGT-3 in the surface sediments of this area (Wang et al., 2015), whereas the increased ratio of GDGT-2 over GDGT-3 in the deep-water column seems to be responsible for a warm bias of TEX_86_-derived temperature in other marine environments (Taylor et al., 2013; Hernandez-Sanchez et al., 2014). In this study, however, no correlation is exhibited between GDGT-2/3 ratio and [MG-II 16S]/[Archaea 16S] ratio (Figure 5I). A recent study by Kim et al. (2015) suggested that coincident increases in GDGT-2 and the crenarchaeol regioisomer and decreases in GDGT-1 and GDGT-3 shifted TEX_86_-derived temperatures toward higher values in the deep-water surface sediments of the Mediterranean Sea. In combination with our data (Figures 4, 5), these observations are consistent with the interpretation that planktonic *Euryarchaeota* have the potential to bias TEX_86_ by changing the distribution of TEX_86_-related GDGTs (especially those with 1–3 cyclopentane rings) in different marine environments.

## Conclusions

This study assessed the relationship between TEX_86_-related GDGTs and MG-II *Euryarchaeota* along a salinity gradient from river water to seawater. The fractional abundance of MG-II *Euryarchaeota* was correlated with %GDGTs with cyclopentane moieties as well as ring index values, implying that MG-II *Euryarchaeota* may contribute ringed GDGTs to the total GDGT pool. This source would thus increase the ring index value and potentially bias the TEX_86_ proxy. However, MG-II *Euryarchaeota* living in the estuary and coastal region did not seem to be a significant source of crenarchaeol. These and other findings based on environmental distributions provide indirect evidence of the lipid profile of MG-II *Euryarchaeota*, which cannot be validated until a pure culture is available.

## Author contributions

JW and CZ designed this study. JW extracted and analyzed lipids. WX extracted and analyzed DNA. JW, WX, YZ, TM, and CZ wrote the paper.

### Conflict of interest statement

The authors declare that the research was conducted in the absence of any commercial or financial relationships that could be construed as a potential conflict of interest. The reviewer FS declared a shared affiliation, with no collaboration, with several of the authors to the handling Editor.

## Supplementary material

Figure S1 Structures of archaeal core GDGTs described in the text.

Figure S2 Relationship of fractional abundances of MG-II *Euryarchaeota* derived from quantitative polymerase chain reaction (qPCR) and pyrosequencing. % MG-II (qPCR) is calculated based on the ratio of MG-II 16S rRNA gene to Archaea 16S rRNA gene; % MG-II (sequencing) refers to the fractional abundance of the MG-II OTUs to the total archaeal OTUs.
