# Supplementary figures and images for: Evaluating Production of Cyclopentyl Tetraethers by Marine Group II Euryarchaeota in the Pearl River Estuary and Coastal South China Sea: Potential Impact on the TEX86 Paleothermometer
Source: Front Microbiol. 2017 Oct 31;8:2077. doi: 10.3389/fmicb.2017.02077 (PMC5671491; doi:10.3389/fmicb.2017.02077)

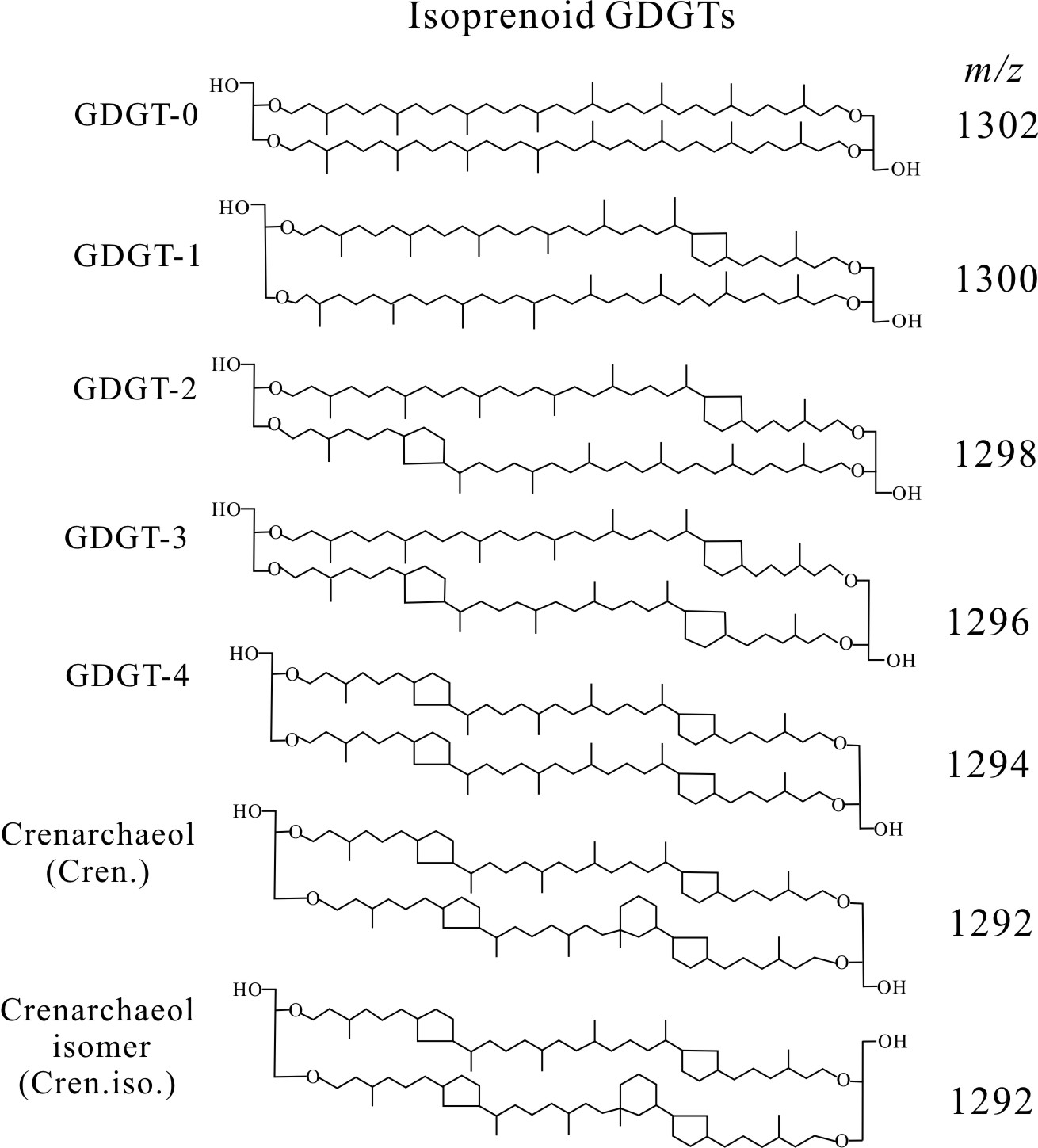

Supplement: Figure S1 — Structures of archaeal core GDGTs described in the text. [file Image1.JPEG]

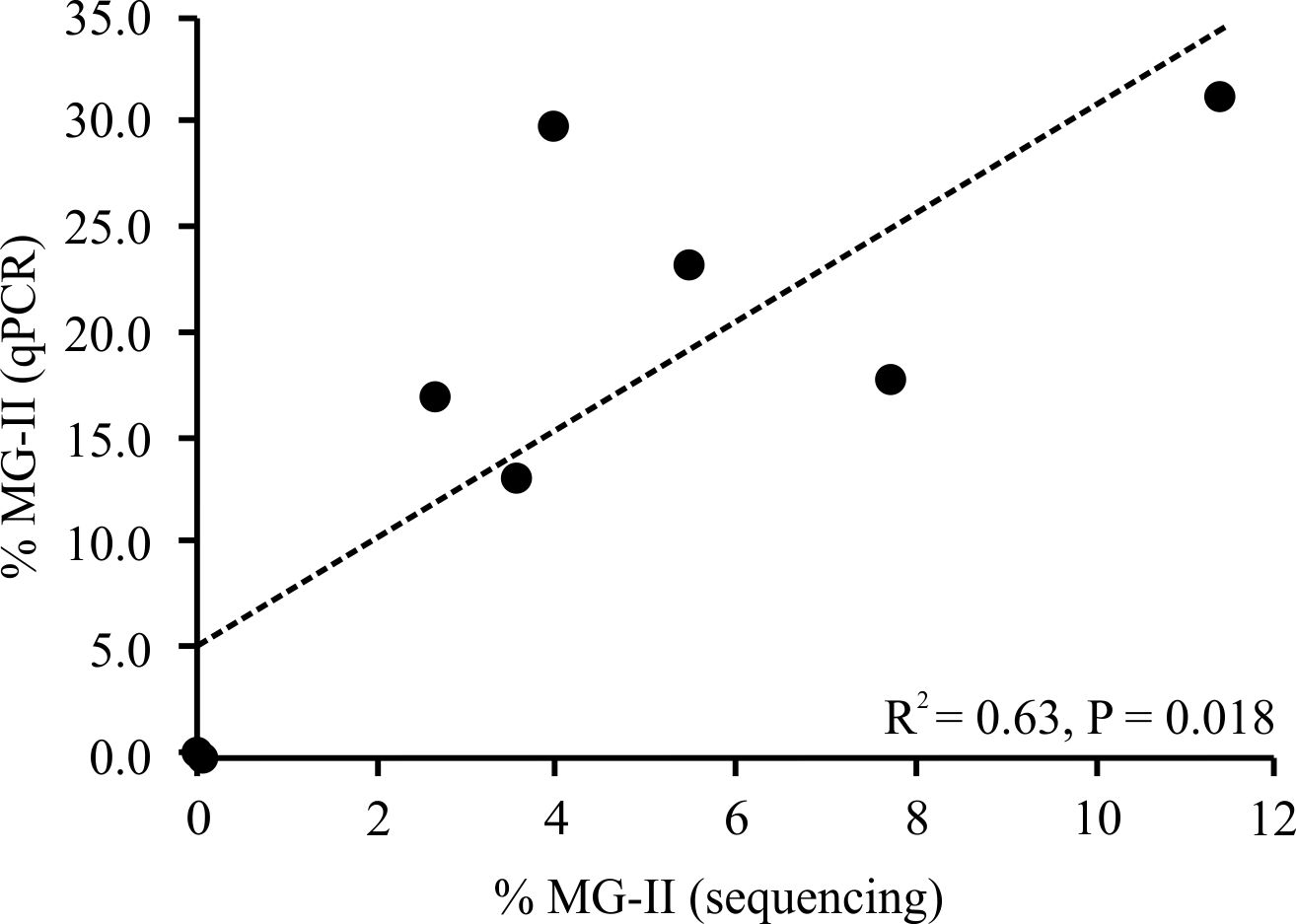

Supplement: Figure S2 — Relationship of fractional abundances of MG-II Euryarchaeota derived from quantitative polymerase chain reaction (qPCR) and pyrosequencing. % MG-II (qPCR) is calculated based on the ratio of MG-II 16S rRNA gene to Archaea 16S rRNA gene; % MG-II (sequencing) refers to the fractional abundance of the MG-II OTUs to the total archaeal OTUs. [file Image2.JPEG]
